# Inflammatory Bowel Disease as a Paradoxical Reaction to Anti-TNF-α Treatment—A Review

**DOI:** 10.3390/life13081779

**Published:** 2023-08-20

**Authors:** Ioana Ruxandra Mihai, Alexandra Maria Burlui, Ioana Irina Rezus, Cătălina Mihai, Luana Andreea Macovei, Anca Cardoneanu, Otilia Gavrilescu, Mihaela Dranga, Elena Rezus

**Affiliations:** 1 Department of Rheumatology and Rehabilitation, Faculty of Medicine, “Grigore T. Popa” University of Medicine and Pharmacy, 700115 Iasi, Romania; luana.macovei@umfiasi.ro (L.A.M.); anca.cardoneanu@umfiasi.ro (A.C.); elena.rezus@umfiasi.ro (E.R.); 2 Department of Dermatovenerology, Faculty of Medicine, “Grigore T. Popa” University of Medicine and Pharmacy, 700115 Iasi, Romania; ioanairinarezus@yahoo.co.uk; 3 Department of Gastroenterology, Faculty of Medicine, “Grigore T. Popa” University of Medicine and Pharmacy, 700115 Iasi, Romania; catalina.mihai@umfiasi.ro (C.M.); otilia.gavrilescu@umfiasi.ro (O.G.); mihaela.dranga@umfiasi.ro (M.D.)

**Keywords:** paradoxical reaction, anti-TNF-α, inflammatory bowel disease, Crohn’s disease, ulcerative colitis, biologic therapy

## Abstract

TNF-α inhibitors (TNFis) have revolutionized the treatment of certain chronic immune-mediated diseases, being widely and successfully used in rheumatic inflammatory diseases, and have also proved their efficacy in the treatment of inflammatory bowel disease (IBD). However, among the side effects of these agents are the so-called paradoxical effects. They can be defined as the appearance or exacerbation of a pathological condition that usually responds to this class of drug while treating a patient for another condition. A wide range of paradoxical effects have been reported including dermatological, intestinal and ophthalmic conditions. The causal mechanism of occurrence may implicate an imbalance of cytokines, but is still not fully understood, and remains a matter of debate. These paradoxical reactions often show improvement on discontinuation of the medication or on switching to another TNFi, but in some cases it is a class effect that could lead to the withdrawal of all anti-TNF agents. Close monitoring of patients treated with TNFis is necessary in order to detect paradoxical reactions. In this study we focus on reviewing IBD occurrence as a paradoxical effect of TNFi therapy in patients with rheumatological diseases (rheumatoid arthritis, psoriatic arthritis, ankylosing spondylitis, and juvenile idiopathic arthritis).

## 1. Introduction

Tumor necrosis factor alpha (TNF-α) is a pro-inflammatory cytokine that induces the activation of inflammatory cells, the production of cytokines, and the expression of adhesion molecules [1]. TNF-α has been shown to be involved in the pathogenesis of several immune-mediated diseases including rheumatoid arthritis (RA), ankylosing spondylitis (AS), juvenile idiopathic arthritis (JIA), psoriatic arthritis (PsA), psoriasis, and inflammatory bowel diseases (IBD) [2]. TNF-α inhibitors (TNFis) have revolutionized the treatment of certain chronic immune-mediated diseases, being widely and successfully used in rheumatic inflammatory diseases such as AS, RA, PsA, and JIA [3,4]. TNFis have also demonstrated their effectiveness in the treatment of IBD, Crohn’s disease (CD), and ulcerative colitis (UC) [5,6,7]. Currently, there are five TNFis; four of them are monoclonal antibodies including infliximab (IFX), adalimumab (ADA), certolizumab pegol (CZP), and golimumab (GOL). The fifth, etanercept (ETN), is a soluble TNF receptor fusion protein consisting of the extracellular portion of the human TNF receptor linked to the Fc portion of human IgG [8,9]. TNF-α inhibitors have a dramatic impact on disease control in the event of a patient being refractory to classic disease-modifying treatments. Their mechanism of action involves binding to TNF and suppressing the immune response [4]. 

**Figure 1:**
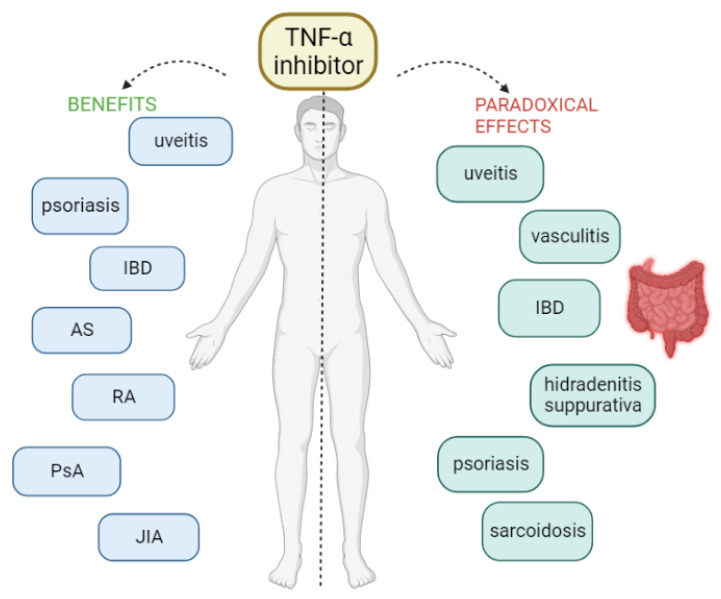
AS = ankylosing spondylitis, IBD = inflammatory bowel disease, JIA = juvenile idiopathic arthritis, PsA = psoriatic arthritis, RA = rheumatoid arthritis.

However, as with most drugs, various adverse reactions have been reported with the use of TNF-α inhibitors, ranging from infections to the risk of malignancy [7,10]. The most common adverse effects of TNFis are mild-to-moderate degrees of itching, pain, swelling, and redness at the site of injection or infusion [2]. In addition to the known adverse effects, some unexpected and rare side effects have been described over the last few years, the so-called paradoxical effects because they appear after the initiation of therapy with an anti-TNF-α that is normally used in their treatment [2]. In other words, these therapies can induce or exacerbate conditions that were intended to be treated. Many and various paradoxical effects have been reported under anti-TNF treatment, including new-onset or exacerbation of the underlying disease. Various organs or tissues can be affected, without being considered as an evolution of the pre-existing condition. Specifically, cases of new-onset or exacerbation of IBD, sarcoidosis and other granulomatous diseases, psoriasis, vasculitis, hidradenitis suppurativa, uveitis, scleritis, vitiligo, alopecia areata, and glomerulonephritis have been reported [2,3,8,9,11] (Figure 1). 

Several case series have reported an increased risk of de novo IBD, mainly CD, under treatment with anti-TNF agents [12]. IFX and ADA are human monoclonal anti-TNF-α antibodies, used in the treatment of CD. IFX has also proven its effectiveness in the treatment of UC [2]. Unlike other anti-TNF-α, ETN has not demonstrated its effectiveness in the treatment of IBD and is more frequently reported with the development of CD or UC in adults and occasionally also in children and adolescents [11,13,14]. 

The present review aims to describe the findings regarding the association between anti-TNF-α treatment and the occurrence of IBD as a paradoxical effect in patients with rheumatological diseases.

## 2. Materials and Methods

A search of the published literature was conducted by exploring PubMed, Google Scholar, EMBASE and MEDLINE databases. The following search terms relating to the key question were set for the search including: “Inflammatory bowel diseases” AND “paradoxical effects” “biologic therapy” AND “paradoxical effects” “ulcerative colitis” AND “paradoxical effects”, “Crohn’s disease” AND “paradoxical effects”, “paradoxical IBD”, “anti-TNF-α/TNFi” AND “paradoxical effects”, “Etanercept” AND “paradoxical effects”, “Adalimumab” AND “paradoxical effects”, “Infliximab” AND “paradoxical effects”, “Golimumab” AND “paradoxical effects”, “Certolizumab” AND “paradoxical effects”. Studies evaluating any possible associations between biologic therapy and IBD as a paradoxical effect were identified. We reviewed the studies published between 2000 and 2023, excluding studies that were in a language other than English. Commonly cited published literature with high-quality research methodology/results and additional articles from bibliographies of recovered papers were examined and included where relevant.

## 3. Results

### 3.1. Paradoxical IBD in Rheumatoid Arthritis

TNF-α is responsible for the synovial inflammation in RA, TNFis being successfully used in the treatment of the disease. Several authors describe the occurrence of IBD as a paradoxical reaction to anti-TNF-α treatment in patients diagnosed with RA (Table 1). The association between inhibition of TNF-α in patients with RA and the onset of IBD is unclear [15]. 

O’ Toole described the occurrence of IBD in nine patients diagnosed with RA, all presenting CD as a subtype of IBD and all being treated with ETN [14]. In O’Toole’s study, however, most of the cases described lacked details regarding radiological or histological evidence of the condition, as well as the time period between the initiation of biological therapy and the appearance of IBD and the follow-up. Krishnan also described 103 cases of IBD that occurred in patients with RA that were treated with anti-TNF-α [15]. Most of them were also treated with ETN (53 cases), 25 patients were under treatment with IFX, 24 with ADA and just 1 patient was treated with GOL. The majority of the patients were female: 80 F vs. 19 M (unknown in 4 cases). UC occurred in 51 cases and CD in 46 cases (unknown in 6 cases). The median age of patients was 51 ± 15 years [15].

Some authors described isolated cases of IBD in RA patients treated with anti-TNF-α [3,4,16,17,18]. UC occurred in two isolated cases, one in a 20-year-old patient treated with ADA and one in a 55-year-old man under treatment with IFX. In both cases, IBD appeared after 4 months of using the anti-TNF-α agent [16,17]. CD occurred in two cases where ETN was being used for a duration of 33 months and 8.5 years, respectively. In both cases, a therapeutic switch to ADA led to a favorable evolution [3,4]. Salazar also reported a case of CD occurrence under ADA treatment, after 2 years of use, and the biological agent was changed to ETN with a favorable evolution as well, with remission of both IBD and RA. In some cases, CS/Mesalazine/Probiotics were added for a better approach in controlling the IBD [18].

### 3.2. Paradoxical IBD in Psoriatic Arthritis

Regarding PsA, only two cases of IBD occurrence as a paradoxical effect of anti-TNF-α therapy have been reported in the literature (Table 2). Toussirot et al. described the case of a 40-year-old woman who developed UC [3], while Oh et al. reported the case of a 21-year-old man who developed CLD [19]. In both cases, the biological agent used was ETN in a dose of 50 mg/week. As a therapeutic approach, in the first case ETN was discontinued and replaced with ADA, while in the second case, ETN was continued and Mesalamine was added, with favorable evolution in both cases.

### 3.3. Paradoxical IBD in Ankylosing Spondylitis

TNF is important in the pathogenesis of AS, since TNF concentrations are elevated in the serum and synovial tissue of these patients [20,21]. TNF appears to be key in the inflammatory response observed in AS [22]. Blocking the pro-inflammatory effects of TNF reduces the symptoms and clinical signs of AS, improving the quality of life. Thus, anti-TNF-α therapy is successfully used in the treatment of AS [23]. However, there are a few studies described in the literature that reported the occurrence of paradoxical effects in patients with AS treated with anti-TNF (Table 3). However, these paradoxical events are rare, and the underlying mechanism is not completely understood.

Braun et al. reviewed the data from nine trials (seven placebo-controlled) of patients with AS treated with anti-TNF agents, evaluating the flares or de novo IBD cases [24]. There were 14 cases of IBD (5 new onsets and 9 flares) reported for ETN: 8 cases of CD (4 new onsets and 4 flares) and 6 cases of UC (1 new onset and 5 flares), corresponding to 2.2 cases of IBD per 100 patient-years of treatment with ETN. There was only one case of IBD (flare of CD) reported for IFX (0.2 per 100 patient-years). For ADA, three cases of IBD (all flares) were reported: 1 CD and 2 UC, corresponding to 2.3 cases of IBD per 100 patient-years with ADA treatment. The relative risk for flare of IBD or development of a new-onset IBD during ETN treatment was determined as 18 times higher than IFX therapy [24].

Uskudar et al. compared the occurrence of IBD in patients with AS treated with anti-TNF and in those without anti-TNF treatment [25]. Seven patients (4.5%) were from the group of those treated with anti-TNF, and three (1.1%) from the group of those treated with non-anti-TNF. The incidence of IBD was 2.4% of patients, all presenting CD as a subtype of the disease. Among the seven patients with new-onset IBD treated with anti-TNF, three were under treatment with ETN, one with IFX and one with ADA. There was no statistically significant difference between ETN and IFX, ETN and ADA or IFX and ADA. After establishing the diagnosis of IBD, a therapeutic switch to another anti-TNF agent was performed in most cases with or without the association of azathioprine (AZA) or CS, with favorable evolution in all seven cases. The independent risk factors for IBD development in AS patients were family history of AS and treatment with anti-TNF agents. In Uskudar’s study, the relative risk of an AS patient experiencing new-onset IBD during anti-TNF treatment was four times higher than those patients receiving drugs, except for anti-TNF agents [25].

In Braun’s study, the frequency of de novo IBD in patients with AS was evaluated at 0.8 per 100 patient-years with ETN and as 0.5 per 100 patient-years with placebo [24]. In Uskudar’s study, the rate was 1.6 per 100 patient-years for ETN, 1.5 per 100 patient-years for IFX, and 0.8 per 100 patient-years in ADA [25]. In Uskudar’s study, the average period of anti-TNF agent administration in patients who experienced new-onset IBD was 15.14 ± 8.5 months (median 12 months) while in Braun’s study the new onset or flare of IBD occurred after a mean of 242 days (range 57–545 days) or 8.1 months of treatment with ETN and 1 year of treatment with IFX [24,25]. 

Another study compared 296 patients with spondyloarthropathy (of which 198 had AS) receiving anti-TNF-α agents (IFX/ADA/ETN) with 112 patients taking disease modifying antirheumatic drugs (DMARDs), to evaluate the paradoxical IBD development [2]. Four new-onset IBD cases were identified in patients treated with anti-TNF drugs (three on ETN with a rate of one per 100 patient-years and one on IFX with a rate of 0.3 per 100 patient-years). No case of IBD as a paradoxical effect was reported under ADA, but the exposure period to ADA was the shortest [2]. 

On the other hand, in the study conducted by Braun, as well as in the one conducted by Fouache, no statistically significant difference was identified in new-onset IBD between patients treated with placebo and those treated with anti-TNF agents [2,24].

Toussirot et al. identified 12 patients with AS who developed IBD under anti-TNF treatment, 10 of whom were under treatment with ETN and 2 with IFX [3]. The most frequent were cases of CD (five patients) or CLD (six patients) and only one case of indeterminate colitis. Patients with AS had pure axial disease in seven cases, and predominantly peripheral arthritis was only noticed in one case. The discontinuation of the offending agent and therapeutic switch led to a favorable evolution in all the described cases [3].

Song et al. also described three patients treated with ETN for active AS who developed a new onset of CD, while AS-related symptoms responded well to ETN [26]. Typical symptoms of active CD occurred 11, 12, and 26 months after the start of ETN therapy. Colonoscopic and histopathological examinations were compatible with CD in all patients. ETN was stopped, and CD responded well to standard treatment. One of the three patients was re-exposed to ETN (+AZA) later on, and he flared 6 months after reinstitution. The two patients without re-exposure to ETN did not have further CD flares [26]. Calin et al. also described two cases of IBD in AS patients, one of CD and one of UC occurrence after an average of 8.1 months of ETN use [27].

Other authors described isolated cases of IBD in patients with AS treated with anti-TNF-alpha [28,29,30,31,32,33,34,35,36,37,38,39]. In almost all described isolated cases, the incriminating agent was ETN and the subtype of IBD was CD. The time period of IBD use varied between 21 weeks and 10 years. Although IFX is used successfully both in the treatment of rheumatological diseases and IBD, there are cases in the literature that reported the occurrence of CD in patients with AS treated with IFX. There were only two isolated cases where CD occurred after treatment with IFX. IBD occurred after 6 and 22 months, respectively, of IFX use. In one case, IFX was continued and Mesalazine was added, with a favorable evolution. It is highly probable that the patient had HLA B27-positive spondyloarthropathy related to his underlying IBD, even though the patient denied gastrointestinal symptoms prior to IFX use [34]. The other case does not mention the treatment followed.

The most common symptoms for which patients presented were abdominal pain and diarrhea. Most of the reported cases were described in male patients, a fact probably explained by the more frequent occurrence of AS in men [40]. IBD as a paradoxical effect usually occurred in young patients, with an age range between 23 and 46 years old. The diagnosis of IBD was established colonoscopically and on biopsy findings. In all the described cases, the evolution was favorable after stopping the treatment with the inducible agent and performing the therapeutic switch to another anti-TNF-α [28,29,30,31,32,33,34,35,36,37,38,39].

Not only cases of de novo IBD were reported, but also flares of the disease. In Braun’s study, more flares of IBD than new onsets of IBD were reported [24]. Marzo-Ortega et al. also reported two cases of patients with AS associated with CD treated with ETN, whose arthritis showed an excellent response with complete resolution of spinal pathology, whereas their CD persisted or flared a short time after initiation of ETN [37]. Prescott also described a flare of IBD in a patient who was diagnosed with AS associated with UC [16]. After 3 to 4 weeks of ETN therapy, the patient developed sudden-onset diarrhea, weight loss, fever, chills, and night sweats. The colonoscopy revealed severe inflammation, with deep ulcers throughout the colon. Microscopic histological examination revealed moderately active UC. Administration of ETN was discontinued. His condition improved rapidly after initiation of prednisone 40 mg daily, which was subsequently tapered completely. Once off ETN, the patient did not have any recurrence of his UC symptoms [16].

Regarding treatment with GOL, data in the literature did not report cases of de novo occurrence of IBD, but several cases of IBD flare were described [38,39]. Bawany reported the case of a 25-year-old man, known to have AS and UC, who developed UC exacerbation after 3 months of GOL use. GOL was stopped, the Mesalamine dose was increased and treatment was switched to ADA [38]. Fiehn also describes three cases of IBD flares after 5 months (two cases) and 2 months, respectively, of GOL use. After GOL discontinuation and CS administration, all cases presented a favorable evolution [39].

**Table 3:** IBD in Ankylosing Spondylitis.

Study, Year	Number of Patients, Sex, Age	Treatment	Interval from Anti-TNF Onset to IBD Onset	IBD	Outcome
Calin et al., 2004 [27]	2 n/a	ETN	8.1 months	1 CD 1 UC	n/a
Braun et al., 2007 [24]	18 F + M	14 ETN	n/a	8 CD 6 UC	n/a
		1IFX		1 CD	
		3 ADA		1CD 2UC	
O’Toole et al., 2016 [14]	14 F + M	14 ETN	n/a	11 CD 3 UC	n/a
Toussirot et al., 2012 [3]	5 F, 7 M Mean age: 42.5	10 ETN 2 IFX	n/a	5 CD 6 CLD 1 ind colitis	All switch to another anti-TNF, all favorable evolution
Song et al., 2008 [26]	3 F + M	3 ETN	11 months; 12 months; 26 months	3 CD	2 controlled with SSZ and CS; One with IFX 2 patients—no flares 1 patient re-exposed to ETN—flare 6 months later
Fouache et al., 2009 [2]	3 F, 1 M Mean age: 38	3 ETN 1 IFX	17 months	IBD n/a	Anti-TNF stopped
Uskudar et al., 2019 [25]	7 F + M Mean age: 41.9 ± 11.6	3 ETN 3 IFX 1 ADA	n/a	7 CD	Switch: 4 ADA 1 CZP 1 IFX + AZA 1 ETN + AZA
Jethwa et al., 2013 [28]	1 M, 45 years	ETN	6 months	CD	Switch to ADA, no flare
Haraoui et al., 2009 [29]	1 M, 26 years	ETN	16 months	CD	IFX + MTX, favorable evolution
Brandt et al., 2004 [30]	1 F, 46 years	ETN	21 weeks	CD	CS + Mesalazine, Favorable evolution
Davis et al., 2003 [23]	1 n/a	ETN	n/a	CD	n/a
Yazisis et al., 2008 [31]	1 M, 23 years	ETN	6 months	CD	CS + SSZ, favorable evolution
Elkayam et al., 2008 [32]	1 M, 33 years	IFX	22 months	CD	IFX continued + Mesalazine, favorable evolution
Baraliakos et al., 2005 [33]	1 F, 28 years	ETN	n/a	CD	ETN discontinued CS + Mesalazine Favorable evolution
Tsochatzis et al., 2007 [34]	1 M, 36 years	IFX	6 months	CD	n/a
Mrabet et al., 2012 [35]	1 M, 27 years	ETN	11 months	CD	IFX Favorable evolution
Tolu S et al., 2018 [36]	1 M, 29 years	ETN	10 years	CD	ADA Favorable evolution
Hutchings et al., 2019 [4]	1 M, 49 years	ETN	8.2 years	CD	ADA + Mesalamine Favorable evolution
Marzo-Ortega et al., 2001 [37]	1 M, 27 years 1 M, 26 years	ETN ETN	10 weeks	CD flare CD flare	n/a
Prescott et al., 2007 [16]	1 M	ETN	3-4 weeks	UC flare	ETN discontinued CS Favorable evolution
Bawany et al., 2014 [38]	1 M, 25 years	GOL	3 months	UC flare	GOL discontinued Mesalamine Switch to ADA Favorable evolution
Fiehn et al., 2011 [39]	1 F, 47 years 1 F, 43 years 1 M, 72 years	3 GOL	5 months 5 months 2 months	2 CD flare 1 UC flare	3 GOL discontinued CS 1 switch to ADA Favorable evolution

F = female, M = male, IBD = Inflammatory Bowel Disease, UC = ulcerative colitis, CD = Crohn’s disease, CLD = Crohn’s-like disease, ind = indetermined, IFX = Infliximab, ETN = Etanercept, ADA = Adalimumab, GOL = Golimumab, CZP = Certolizumab pegol, n/a = not available, CS = corticosteroids, TNF-α = tumor necrosis factor alpha, 5-ASA = 5-aminosalicylates, SSZ = Sulfasalazine, AZA = Azathioprine, MTX = Methotrexate.

### 3.4. Paradoxical IBD in Juvenile Idiopathic Arthritis

JIA is one of the most common rheumatological diseases in children. The prevalence varies between 16 and 150 per 100,000 children [41]. The treatment of the disease aims at decreasing disease activity, the most-used therapies being NSAIDs, steroids, conventional synthetic (cs)DMARDs and biological (b)DMARDs [42]. Gastrointestinal (GI) disease appears to be one of the documented extra-articular manifestations of JIA [43,44,45]. On the other hand, 16–33% of children with IBD experience joint involvement over the course of the illness [43,44,45]. IBD is a rare comorbidity of JIA, with its subtypes: UC, CD or indetermined IBD [46]. While in the general population the incidence of IBD is approximately 0.23/1000 person-years [47], Barthel reported an incidence of 1.31/1000 patient-years for IBD in a registry of 3071 JIA patients treated with and without bDMARDs [48]. IBD incidence in JIA patients ranges from 20 to >40 times the IBD rates in the general pediatric population [48,49,50]. There have also been cases described in the literature where IBD occurred as a paradoxical effect to TNFi treatment (Table 4).

There is an increased interest regarding IBD in patients with JIA, since cases have been described of IBD onset upon treatment with anti-TNF-α, especially ETN. In general, ETN offers an acceptable safety profile in children with JIA, and provides significant and sustainable improvement in disease manifestations [50]. According to recent data, 1.9 new IBD cases occurred per 100 JIA patient-years of ETN [51]. In almost all cases described in the literature, the incriminating agent was ETN. In the study by Van Dijken et al., 13 cases of IBD (9 CD, 3 UC) were identified in patients with JIA under ETN treatment [50]. Gerloni et al. reported the adverse events seen in a cohort of 163 patients with JIA treated with IFX (68 patients) or ETN (95 patients) [51]. IBD was found in five patients treated with ETN, while no such case was reported in patients treated with IFX [51]. Tarkainen conducted a study on a group of 292 patients diagnosed with JIA, identifying four cases of IBD, of which three were undergoing treatment with ETN and one patient was receiving treatment with IFX [52]. In Van Straalen’s study, out of 27 patients with known onset of IBD, most—13 (48.1%)—used ETN (with or without MTX) [53]. Dallochio also described the occurrence of IBD in eight patients with JIA treated with ETN [49]. Also, in another study that identified 28 JIA patients who developed IBD, the majority (23 patients—82.1%) received treatment with ETN [54]. ETN alone was associated with an increased incidence of IBD [54]. Van Straalen et al. observed that incidence rates of IBD were significantly higher for combination therapy with ETN and MTX, ETN monotherapy and IFX compared with MTX monotherapy. No significant difference was found for ADA therapy [53].

MTX proved to be protective against IBD in JIA [55]. In Brokaert’s study, the incidence of IBD was lower in patients treated with MTX, but higher in patients treated with ETN, except if ETN was combined with MTX [54]. In Barthel’s study, the IBD incidence was also significantly lower in patients treated with MTX or with an association of ETN and MTX, compared with patients not treated with MTX [48]. However, Van Straalen et al. observed that ETN was associated with IBD in JIA, regardless of concomitant use of MTX [53].

Regarding the IBD subtype, most studies have described the predominant occurrence of CD. Van Straalen conducted a study on a group of 8942 patients, identifying 48 (0.54%) cases of IBD: these included 13 cases (27%) of UC, 22 cases (46%) of CD and 13 cases (27%) of indeterminate colitis [53]. Dallochio identified five patients with CD and three with indeterminate IBD [49]. In another study, 82.1% of patients presented with CD [54]. Also, in Gerloni and Barthel’s studies, CD stood out more frequently than UC [48,51].

In the published studies, the interval of IBD onset from the initiation of anti-TNF therapy varied. Dallochio described patients being treated with the TNFi for 7–78 months, while in Van Dijken’s study the interval varied between 9 days and 4.5 years [49,50]. In another study, IBD occurred in an average of 382 days [53]. The median age of IBD onset varied between 4 years and 17 years. 

Van Straalen observed that patients who developed IBD were significantly more often male, HLA-B27 positive and older at JIA onset than patients who did not develop IBD [53]. On the other hand, Barthel concluded that there was no significant difference in sex, HLA-B27 positivity, or ANA positivity [48]. Furthermore, in Van Straalen’s study, patients had significantly more often a family history of autoimmune disease(s)—(psoriasis, RA and Hashimoto’s thyroiditis) [53]. Also, the incidence rates of IBD regarding drug therapy in ERA patients were higher, compared with the total cohort. Other ILAR categories, ANA status and RF status did not differ significantly between IBD and non-IBD patients [53]. Also, in Barthel’s study, patients with IBD more commonly had ERA, extended oligoarthritis, psoriatic arthritis, and also rheumatoid factor (RF)-negative polyarthritis. No IBD occurred in patients with systemic JIA or RF-positive polyarthritis [48].

Patients had never previously suffered from abdominal complaints, and they did not present other signs, suggesting that their arthritis could be a complication of a pre-existing subclinical IBD. The most common symptoms included abdominal pain, diarrhea, blood in stools, anorexia, fever and weight loss. The diagnosis was established on colonoscopy and biopsy findings. Clinical remission of IBD was obtained in all patients after discontinuation of ETN and initiation of IBD-specific therapy (including therapeutic switch to another TNFi) [48,49,50,51,52,53,54,55,56,57,58,59,60].

**Table 4:** Paradoxical IBD in Juvenile Idiopathic Arthritis.

Study, Year	Number of Patients, Sex	JIA Onset	IBD Onset	Interval Onset JIA to IBD	Interval Onset Anti TNF to IBD	JIA Subtype	IBD	HLA-B27	Treatment	Outcome
Gerloni et al., 2008 [51]	5 n/a	n/a	n/a	n/a	n/a	n/a	2 CD 1 ind IBD	n/a	ETN	All switch to another anti-TNF
Dallochio et al., 2010 [49]	6 F 2 M	3–13 years	n/a	n/a	7–78 months	4 oligo JIA 1 RF poly JIA 1 systemic JIA 2 ERA	5 CD 3 ind IBD	n/a	8 ETN	All ETN discontinued 6 IFX 2 AZA 3 + Mesalazine
Van Dijken et al., 2011 [50]	10 F 3 M	1–16 years	Median: 12 years	5 years and 3 months	9 days–4.5 years	4 poly JIA 5 oligo-ext JIA 2 ERA 2 systemic JIA	9 CD 3 UC 1 ind IBD	All−	ETN	8 switch to IFX 2 switch to ADA 3 Other (CS/SSZ/Mesalazine/Pentasa) ±other
Tarkiainen et al., 2011 [52]	F	9.8 years	15.2 years	5.4 years	2.1 years	Seronegative Poly	UC	+	ETN	ETN + Mesalazine + CS
	M	9.0 years	12.6 years	3.6 years	2.8 years	ERA	CD	+	ETN	ETN + Mesalazine
	F	4.3 years	14.8 years	10.5 years	4.4 years	Ind arthritis	UC	+	ETN	ETN + SSZ + CS
	F	3,7 years	13.3 years	9.6 years	1.4 years	Seronegative poly	CD	−	IFX	AZA + CS + switch to ETN
Toussirot et al., 2012 [3]	1 M	17 years	n/a	6 years	n/a	ERA	CD	n/a	ETN	Switch to ADA
	1 M	11 years		1 year		ERA				Switch to IFX
Barthel et al., 2015 [48]	3 M 8 F	6.1 ± 3.9 years	13.4 ± 3.4 years	7.2 ± 4.0 years	1.71 years	3 oligo-ext 4 seroneg polyarthritis 2 ERA 2 psoriatic JIA	8 CD 3 UC	2 HLA B27+ 7 ANA+	9 ETN ± CS/NSAIDs/csDMARDs 2 Other (SSZ, MTX, LEF)	ETN stopped Standard care (N/A) Switch: 4 ADA, 1 IFX
Van Straalen et al., 2022 [53]	48 IBD (27 known onset) n/a	n/a	13.7 years	n/a	382 days	n/a	22 CD 13 UC 13 ind	n/a	13/27 ETN	n/a
Broekaert et al., 2023 [54]	28 n/a	n/a	n/a	n/a	n/a	25% ERA	23 CD 4 UC 1 ind IBD	20.3%+	23 ETN 5 other (NSAIDs, CS, MTX, SSL, LEF)	n/a
Wiegering et al., 2010 [11]	1 F	7 years	11 years	4 years	1 year	Oligo JIA (ANA+, RF−)	CD	-	ETN	SSZ—inefficient. then ADA
Flemming et al., 2013 [56]	1 M	12 years	14 years	2 years	4 months	ERA (ANA, RF−)	CD	-	ETN	ETN stopped, switch to IFX
Ruemmele et al., 2004 [57]	1 M	2.5 years	6 years	3.5 years	n/a	Oligo JIA (ANA+, RF−)	CD flare	n/a	ETN	ETN stopped + 5-ASA then AZA then switch to IFX
Oikonomou et al., 2010 [58]	1 F	2 years	17 years	15 years	n/a	Oligo JIA	CD	n/a	ETN	ETN stopped Switch to IFX and then ADA
Actis et al., 2012 [59]	1 M	8 years	13 years	5 years	28 months	Oligo-ext JIA	1 UC	n/a	ETN	Switch to ADA then IFX Mesalamine—CS—AZA
Zeits et al., 2015 [60]	1 M	12 years	n/a	n/a	2 months after ADA	n/a	CD	+	ETN then ADA	ADA Continued + CS then switch to IFX then right-sided colectomy with an ileocolic anastomosis then ETN—then stop ETN

F = female, M = male, IBD = Inflammatory Bowel Disease, UC = ulcerative colitis, CD = Crohn’s disease, ind = indetermined, IFX = Infliximab, ETN = Etanercept, ADA = Adalimumab, poly = polyarticular JIA; oligo = oligoarticular JIA; oligo-ext = oligo-extended JIA; ERA = enthesitis-related arthritis, MTX = metotrexate, LEF = leflunomide, AZA = azathioprine, 5-ASA = 5-aminosalicylates, SSZ = sulfasalazine, CS = corticosteroids, NSAIDs = nonsteroidal anti-inflammatory drugs, ANA = antinuclear antibodies, RF = rheumatoid factor, n/a = not available.

## 4. Discussion

Many reports indicate that during periods of intense immunosuppression with anti-TNF-α, IBD may develop or worsen while patients are taking the drugs used to treat these disorders. Thus, TNF inhibition seems to have paradoxical pro-inflammatory effects, in addition to the known anti-inflammatory ones. Increased numbers of unexpected paradoxical events involving TNFi therapy have been described, with an estimated incidence of more than 10% in patients receiving a TNFi [48,61]. Perez de Lis et al. showed that IBD induced by biologics was the second most frequent paradoxical reaction developed in rheumatic patients (845 cases of 12.731) [62]. According to Penso, there are no differences in the clinical, endoscopic, or histopathologic characteristics of traditional IBD and paradoxical IBD [63]. Cases have been reported in association with ETN, IFX, and ADA and with underlying conditions such as AS, RA, JIA, and PsA [4]. The underlying disease is usually well controlled by anti-TNF treatment, giving no argument for an eventual flare of the systemic condition to explain the occurrence of the new clinical feature [9].

Several controlled trials and sizable post-marketing studies describing paradoxical effects have been published recently, despite the fact that the majority of instances originate from retrospective research and individual case reports (probably due to the extremely low prevalence of these processes caused) [63]. Evidence shows that most likely, a certain genetic background that favors paradoxical effect development may have a significant influence. This may also help to explain the reported exacerbations of autoimmune conditions in patients exposed to biologics [63]. 

There are few data available regarding the possible predisposing factors that could have precipitated the appearance of IBD under anti-TNF-α treatment. There are different mechanisms that may be involved in the pathogenesis [56,64,65,66]. Paradoxical side effects of anti-TNF medications may be caused by immune-mediated processes [9]. This exact mechanism of damage is not yet known. IBD flares may be associated with immunomodulation, although it is possible that these flares were only coincidental and may not be related to TNFi treatment [16]. The few information that is currently available suggests that TNF inhibition activates autoreactive T cells, which then results in tissue destruction via an autoimmune process [25]. Potential pathophysiological hypotheses include that the introduction of TNF alpha blockers may alter the cytokine balance in patients with a genetic predisposition, having genetic factors like NOD2/CARD15 gene mutations, which may result in background conditions for the development of IBD [3]. Paradoxical effects may be more common in people with these gene variations [11,67]. Toussirot and Wiegering each identified one patient with the NOD2/CARD15 gene mutation, although it is unknown whether this mutation is relevant to the condition, or represents a predisposition to CD [3,11]. However, genetic testing in patients with rheumatic diseases for mutation in IBD-related genes is not routinely necessary, because a clear association is only obvious in a minority of cases [3]. 

Prescott postulated the possibility of the occurrence of IBD as a result of the imbalance between TNF and IFN in susceptible individuals [16]. ETN’s ability to inhibit TNF has been shown to increase T cell production of IFN gamma (IFNg) and TNF [66]. One study found that after RA patients had treatment with ETN, T cells activated by microbial antigens generated more IFN-γ [64]. Thus, increased levels of IFNg and TNF in the intestinal mucosa can trigger IBD in genetically susceptible individuals [19,67]. 

IBD may be influenced by immune system dysregulation directed against microbial antigens located in the intestinal lumen. Increased T cell reactivity to microbial antigens or the inability of regulatory T cells to control normal responses may be the trigger for the disease onset [68]. Cells in the intestine’s natural mucosal immune system stop T cells that are responsive to the bacterial flora from inducing damaging immune reactions. Oh et al. observed that blockade of TNF-α by neutralizing antibodies has inhibitory effects on these regulatory T cells [19]. 

Proof of an association of IBD with TNF treatment is strongest for ETN. O’Toole et al. performed a study on IBD provoked by ETN. They identified 443 cases of de novo onset of IBD and 43 cases (31 CD, 12 UC) of flares of existing IBD reported in association with ETN therapy [14]. A total of 44 cases (41 CD and 3 UC) of de novo IBD were most clearly associated with ETN treatment. Another 382 cases developed IBD after the initiation of ETN therapy, but data provided were inadequate to assess fully causality, although it is considered that in these cases there was a direct relationship between the initiation of ETN and the development of IBD [14]. Hutchings et al. studied the occurrence of paradoxical effects in patients treated with anti-TNF-α, comparing them with a group of patients with rheumatological damage receiving treatments other than anti-TNF-α [4]. He observed paradoxical reactions in three out of nine patients under anti-TNF-α (33.3%), all of whom were under treatment with ETN [4]. Toussirot et al. conducted a study over a period of 2 years, identifying 16 cases of IBD associated with anti-TNF-α treatment, and most of them (14 patients) received ETN [3]. Pérez-De-Lis et al. have researched the BIOGEAS registry that was designed to collect and analyze all the reported data on autoimmune diseases developed in patients exposed to biologics [63]. Among the biological therapies administered, the majority were TNF-targeted therapies, in 716 (85%) cases, and were mainly ETN, in 648 cases [64]. Furthermore, in Krishnan’s study regarding the occurrence of IBD in patients with RA or JIA, most of the patients were also under treatment with ETN (50 patients—90.9% JRA, 53 patients—51.5% RA), and they found a moderately strong association between ETN use and IBD development in juvenile arthritis [15]. Van Dijken et al. calculated a 43-fold increased risk of IBD in patients with JIA treated with ETN, compared to the control population [50].

ETN, in particular, is the main TNF-α inhibitor associated with the development of paradoxical IBD [3,24,36] in adults and occasionally also in children and adolescents [11,69]. This association, which is not incidental, is rare, and is seen especially in patients with spondyloarthritis [3,24,36]. The occurrence of these paradoxical events more frequently under ETN therapy could be explained by the structural and functional differences between the anti-TNF agents [70]. IFX is a monoclonal antibody to TNFα made up of a chimeric protein that directly inhibits the action of TNFα and can bind to cells expressing TNFα in membrane-bound form [71]. ETN, by contrast, is a fully human, genetically engineered fusion protein consisting of two identical chains of the recombinant human soluble receptor TNFR p75 monomer fused with the Fc domain of human IgG1, which binds and inactivates TNFα and lymphotoxin [31,71,72]. ETN has profound effects on inducing and maintaining remission in rheumatological conditions, but is less efficacious in granulomatous disease [70]. ETN did not prove to be efficient in IBD treatment [13,29,73]. IFX is able to induce apoptosis of activated lymphocytes, whereas ETN does not [73]. IFX has been shown to neutralize both soluble and membrane-bound TNF-α (expressed in macrophages and activated T cells in inflamed human gut) with activation of apoptosis of T cells through a caspase-dependent pathway [73], whereas ETN neutralizes soluble TNF-α that does not activate T cell apoptosis, due to the lower binding affinity and inability to cross-fix membrane-bound TNF, due to its monomeric structure [31,73]. IFX and ADA lead to apoptosis of T cells in the lamina propria, while ETN leads to cytokine production, which includes TNF-α and IFN-γ [48]. Binding of TNF-α to ETN prolongs the plasma half-life of the cytokine. In other words, ETN’s neutralization of soluble TNF may generate an increase in cytokines that act in a counter-regulatory way, in part because T cells themselves are “unaffected” by ETN. Regarding ETN therapy, increased counter-regulatory cytokine production may hasten the start of CD [9,51,66,69]. These factors may favor the inflammation in the bowel mucosa and may result in granuloma formation, and thus lead to the development of new-onset IBD [66,73]. Another explanation may be the implication of the IL-23/IL-17 axis in the development of CD [9,74].

Overall, the numerous case reports and case series describing new-onset IBD caused by ETN firmly indicate that ETN rarely induces IBD in susceptible patients. It is tempting to hypothesize that ETN, which has not been shown to have therapeutic benefits for CD, may result in evident mucosal inflammation more frequently than IFX or ADA, which are both very successful treatments for IBD. However, paradoxical induction of IBD by IFX and ADA is also likely, even though the evidence is weaker. 

The gastrointestinal reaction induced by biologics is considered to be uncommon, affecting more frequently the populations with AS, PsA or psoriasis, as an extra-articular manifestation of these diseases [24]. It is known that patients with IBD often have other immune-mediated inflammatory diseases (IMIDs), and the prevalence of any IMID is higher in IBD patients than in the general population. IBD and other autoimmune disorders have been linked in extensive research, particularly AS, which shares clinical, pathological, and genetic characteristics with IBD [75]. Subclinical lesions resembling CD are seen in up to 50% of AS patients [76]. Paradoxical inflammatory conditions may represent the “unmasking” of an underlying inflammatory disease process in susceptible individuals [29]. However, in most of the cases described, there was no clinical evidence of an underlying IBD before the initiation of TNFi therapy. Therefore, two hypotheses can be advanced to explain these events: either the occurrence of IBD is due to the association between IMIDs, or a complication is induced by the anti-TNF agent. This remains an intriguing question that needs further investigation. Data show that anti-TNF medications are likely to exacerbate IBD in people with AS who have a hereditary predisposition to the disease [25]. The likelihood of developing CD or UC is significantly increased (four times higher) in AS or PsA patients [62]; however, the relationship between these immunological diseases is unclear [77]. Tolu et al. estimated that the prevalence of new-onset IBD under TNFi in AS patients was around 0.15%, and the incidence was estimated at 2.2/100 patient-years with ETN and 0.2/100 patient-years with IFX [36]. Additionally, AS patients with a history of IBD had a ten-fold risk increase for developing IBD flares during treatment with ETN [36].

Regarding the IBD subtype, CD was most frequently identified [2,3,4,24]. Several case series have suggested an elevated risk of de novo IBD, mainly CD, under treatment with anti-TNF agents, with an incidence of 1.9 per 100 patient-years [12,50,78]. O’Toole collected data from 49 patients, 44 of them being diagnosed with CD. De novo IBD was more commonly associated with CD than with UC, but no specific CD phenotype was identified [14]. In Toussirot’s study, IBD was classified as typical CD in eight cases, CLD in six cases, indeterminate in one case, and definite UC in one case [3]. Perez de Lis et al. identified induced IBD consisting of CD in 355 cases and UC in 228 cases [63]. Also, in Krishnan’s study, the most frequent subtype of IBD was CD, in 71 cases (44.9%), while UC appeared in 58 cases (36.7%), and the subtype of IBD was not specified in 29 cases (18.4%) [15]. Also, in the isolated cases described by the authors, CD was most frequently identified. It is unknown why TNF inhibition would trigger mainly CD or unclassified colitis [60].

The average age of diagnosis and the time interval between the initiation of anti-TNF treatment and the onset of IBD varies a lot in the data from the literature. In O’Toole et al.’s study, the average age was 38.4 (range 10–68 years) [14]. In Toussirot’s study, the mean age was quite similar, of 41.5 ± 17.4 years [3]. The average duration of treatment prior to IBD development was 3.58 months (range 1–132 ms) in O’Toole’s findings [14], while Toussirot described a higher average duration of treatment prior to IBD development: 29.3 ± 20.1 months [3]. In Krishnan’s study conducted on JIA patients, the time interval varied between 9 days and 4.5 years [15]. Uskudar noted an average duration of treatment of 15.14 months (range 6–30 months)—a significantly shorter treatment period than the treatment period of patients who were on anti-TNF treatment but did not develop IBD [25]. Hutchings et al. estimated that TNFi-induced paradoxical gastrointestinal reactions (new-onset IBD or IBD flares) appear after approximately 4–40 months of treatment (with a median value of 27 months) [4].

NSAIDs are frequently prescribed to patients with rheumatic diseases for their analgesic and anti-inflammatory effects, but they can also have an impact on the entire gastrointestinal system. The damage to the gastrointestinal system might create similarities in endoscopic and pathological characteristics with IBD. However, the absence of concentric diaphragmatic strictures and the presence of cobblestoning, longitudinal ulcers, or inflammatory polyps, along with histologic findings of granulomas, crypt abscesses, or crypt distortion, should indicate CD as the likely cause rather than NSAIDs [79]. Even though a few studies lack information regarding the use of NSAIDs, in most cases reported, the patients stopped using NSAIDs when biologic therapy was initiated or NSAIDs had never been regularly prescribed. In patients that used NSAIDs in association with TNFis, the decision of IBD instead of NSAID-induced enteropathy was made according to the endoscopic and pathologic findings mentioned above.

Regarding the therapeutical approach, these paradoxical reactions often show improvement on discontinuation of the medication or on switching to another anti-TNF agent. If symptoms persist or worsen even upon treatment with a second inhibitor, alternative treatment options should be pursued [60]. The management of IBD cases triggered by the TNFi is largely identical to that of classical IBD cases, and in addition to discontinuation of the offending drug, standard IBD treatment should be applied [60]. In some rare cases, the anti-TNF may be maintained without change, pending additional treatment of IBD with Mesalazine/CS/antibiotics. Most patients in the identified studies had a favorable evolution. In some cases, surgical resection was required to control the IBD and underlying dermatological or rheumatological disease [3].

Limitations of our study consist in its retrospective design, the majority of instances originating from individual case reports (probably due to the extremely low prevalence of these processes caused). For this reason, we cannot have exact data regarding the incidence and prevalence of IBD in rheumatic patients treated with the TNFi. Furthermore, most cases reported lacked adequate detail for understanding the associated risk factors and natural history in greater detail. It is also plausible that these flares could be mere coincidences, and might not be directly connected to TNFi treatment, as no rechallenge was conducted. The presence of a flare upon reattempting the TNF inhibitor treatment would undoubtedly offer more compelling evidence to establish a causal relationship between the TNFi and the disease’s onset. Also, another limitation is bias in reporting of cases, and we cannot exclude the possibility that some cases were not declared and thus missed. However, we tried to collect all the data from the literature on this subject. Looking ahead, an ideal approach would involve the aggregation of a substantial number of IBD cases from diverse national and international cohorts or registries. By doing so, we could perform a comprehensive analysis utilizing multivariable techniques in a case-control study to confirm associations with risk factors, including drug therapy. This more extensive and rigorous investigation would enhance our understanding of the relationships between these factors and IBD, leading to more robust and reliable conclusions. 

## 5. Conclusions

Data from the literature suggest a possible link between the use of anti-TNF-α and IBD occurrence, as a paradoxical effect to these therapies in patients with genetic predisposition. However, the underlying mechanism is still not clear. Most of the reported cases or series of cases concern the occurrence of IBD in patients diagnosed with AS or JIA, the most frequent incriminating agent being ETN. As a subtype of IBD, CD may be more common. Further studies are needed to elucidate the complex association between the occurrence of paradoxical reactions and the use of biologic therapies.

## Figures and Tables

**Table 1:** Paradoxical IBD in rheumatoid arthritis.

Study, Year	Number of Patients, Sex	Age/Mean Age	Treatment	Interval from Anti-TNF Onset to IBD Onset	IBD	RF, CCP Antibodies	Colonoscopy	Biopsy	Outcome
O’Toole et al., 2016 [14]	9 F + M	n/a	9 ETN	n/a	9 CD	n/a	n/a	n/a	n/a
Krishnan et al., 2015 [15]	80 F 19 M 4 n/a	51 ± 15 years	24 ADA 53 ETN 25 IFX 1 GOL	n/a	46 CD 51 UC 6 n/a	n/a	n/a	n/a	50 stopped current anti-TNF-α 33 continued current anti-TNF-α 20 unknown ±5-ASA, CS, antibiotics, ADA/IFX
Prescott et al., 2007 [16]	1 M	55 years	IFX	4 months	UC	RF+ anti CCP+	Moderately congested, erythematosus, friable and granular mucosa in the rectum, sigmoid, splenic flexure, and distal transverse colon	Chronic active colitis with acute cryptitis, crypt abscesses, architectural distortion, dense lymphoplasmacytic lamina propria infiltrate and lymphoid hyperplasia	IFX stopped CS+ Mesalamine
Tursi et al., 2008 [17]	1 F	20 years	ADA 40 mg/2 week + MTX 25 mg/week	4 months	UC	n/a	Diffuse loss of vascular pattern, edema in the mucosa, and diffuse erosions from the rectum to the splenic flexure in a continuous fashion	Cryptic abscesses, a decreased number of goblet cells, and a marked infiltration of neutrophils and lymphocytes	CS Mesalazine Probiotics
Salazar et al., 2013 [18]	1 F	37 years	ADA 40 mg/2 week	2 years	CD	n/a	Disperse and deep ulceration in right colon and lesser ulcers and erythema in rectum and a normal ileum	Compatible with CD	CS Switch to ETN Favorable outcome
Tousirrot et al., 2012 [3]	1 F	83 years	ETN 25 mg/week	33 months	CD	RF− Anti CCP+	Ileum stenosis	Mucosal inflammation with the presence of epithelioid granuloma	ETN discontinued switch to ADA Favorable outcome
Hutchings et al., 2019 [4]	1 F	30 years	ETN	8.5 years	CD	n/a	n/a	Active inflammation, architectural distortion, and pyloric gland metaplasia	Switch to ADA Favorable outcome

F = female, M = male, UC = ulcerative colitis, CD = Crohn’s disease, IFX = Infliximab, ETN = Etanercept, ADA = Adalimumab, GOL = Golimumab, RF = rheumatoid factor, CCP = cyclic citrullinated peptide antibody, CS = corticosteroids, TNF-α = tumor necrosis factor alpha, 5-ASA = 5-aminosalicylates, n/a = not available.

**Table 2:** Paradoxical IBD in Psoriatic Arthritis.

Study, Year	Sex, Age	Treatment	Colonoscopy	Biopsy	IBD	Outcome
Toussirot et al., 2012 [3]	1 F, 40 years	ETN 50 mg/week	Mucosal ulcerations in colon and rectum pancolitis	Superficial ulcerations, cryptic abscesses, distorsion of crypt architecture, no epitheliod granuloma	UC	ETN discontinued and replaced with ADA with favorable intestinal outcome
Oh et al., 2005 [19]	1 M, 21 years	ETN 50 mg/week	Deep ulcerations in the terminal ileum, deep ulcerations with cobblestoning in the cecum, and scattered aphthous ulcers extending from the rectum to the right colon	Areas of acute and chronic inflammation; some crypt destruction, but no transmural inflammation or granulomas were seen, as the biopsies were superficial	CLD	ETN continued + Mesalamine. He was offered IFX to treat both his PsA and CD but he declined

F = female, ETN = Etanercept, ADA = Adalimumab, UC = ulcerative colitis, CLD = Crohn’s-like disease, PsA = psoriatic arthritis.

## Data Availability

The data presented in this study are openly available in its references.
